# Tailoring Momentum and Information Transfer of Structured Light to Adapted Liquid Crystals

**DOI:** 10.1021/acsomega.5c09134

**Published:** 2025-12-01

**Authors:** Silvia Hofmann, Peter Lemmens, Angela Möller

**Affiliations:** † Institute for Condensed Matter Physics, 26527 University of Technology Braunschweig, D-38106 Braunschweig, Germany; ‡ Department of Chemistry, 9182 JGU Mainz, D-55128 Mainz, Germany

## Abstract

We report on an experimental assessment of the generally weak coupling of the electromagnetic phases of structured light to matter. Using a systematic study of a liquid crystal series including plasmonic particles, we optimized the reflectivity of the chiral phase, leading to a high sensitivity and discrimination of orbital angular momenta states of light. The involved inelastic scattering shows a decisive dependence of its energy on scattering vectors in comparing different scattering geometries (transmission and backscattering), and of intensity on fine-tuning the ratio of cholesteryl nonanoate (CN) doped with cholesteryl chloride (CC), CN:*x*CC. We provide spectroscopic evidence for an important role of geometric resonances of the light wavelength with the chiral pitch length and propagation vector, *p*(*T*, *x*), of the liquid crystal. We show that inelastic scattering originates from an exchange process, which is identified by momentum transfer. Tailoring the materials, adding plasmonic effects, and optimizing the laser wavelength, we managed to enhance the low energy response by 42 and 71%, respectively. The role of long-range dipolar interactions as well as geometric and electronic resonances are discussed to dominate these processes and their characteristic energy scales. Our work adds an interesting aspect to potential applications of liquid crystals in advanced photonics such as relevance for enhanced information transfer and communication.

## Graphical abstract

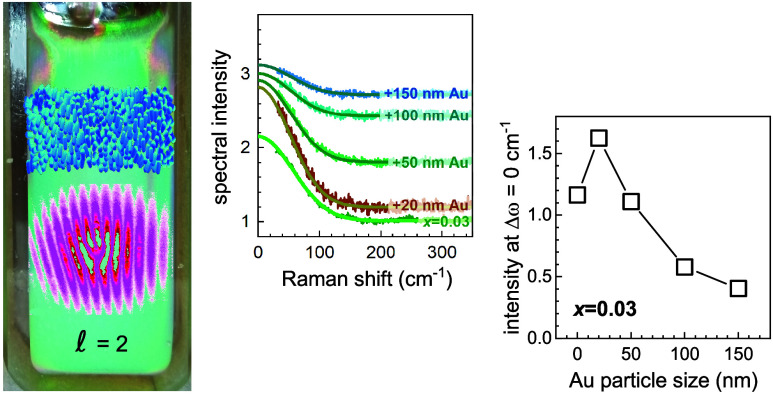

## Introduction

Chirality is an essential property within physics, chemistry, and biology and provides insights into the relation of symmetry and fundamental properties. Due to its nonlocal nature, chirality is directly related to topology and important questions regarding the occurrence of phase factors in natural sciences.^1–3^ Structured, vectorial, or vortex light on the other hand carries orbital (OAM) and spin angular momentum (SAM) based on the chirality of its phase front and helicity of the photons.^4^ It is an exceptional tool to study all aspects of chirality and its relevance has been proven for quantum optics,^5–7^ plasmonics,^8–11^ and manipulation of chiral states of matter. Numerous applications exist,^12^ e.g., in superresolution OAM holography,^13^ separation of enantiomers,^14^ manipulation of Bose–Einstein condensates,^15,16^ and quantum dots.^17,18^ We highlight the potential of structured light for information transfer and enhanced (quantum) communication. This is due to the larger number of degrees of freedom leading to higher transfer rates that the phase front of light can carry compared with conventional polarization states. These degrees of freedom are assigned to the orbital angular momentum (OAM) that is quantized with lℏ per photon, with the topological charge l in the azimuthal phase $e^{\mathrm{il}\varphi }$ that may vary in a much larger range, – ∞≤l≤−∞ . Structured light also allows higher stability in turbulent and strongly fluctuating media.^19,20^

Any substantiation and quantification of information transfer and interaction processes require a fundamental understanding of the microscopic coupling and scattering of structured light to matter. This includes several orders of magnitude in length, energy scales, and involves interaction terms which are far from being trivial.^21^ Therefore, we need experimental studies that span a larger range of different interaction parameters that vary thermal fluctuations, local energy density, and resonance conditions, as discussed below.

In this work, we will elaborate on the question under which conditions the coupling of light with OAM and SAM to matter can be maximized.^22,23^ This question is closely related to locally impinging novel states of matter using structured light. Reflectivity of light and, as a higher order scattering process, Raman scattering (RS) as a function of the OAM and SAM are obvious experimental probes. RS is valuable as its processes involve high energy virtual states. Raman optical activity relates to the characterization of chiral matter. With respect to information transfer and manipulation of matter,^24^ it is useful to view this process as a transfer of angular and translation momentum. We define a coupling strength by the intensity of inelastic scattered light as a function of the polarization of the states. Studying a feasible temperature dependence elucidates the thermodynamic statistics of such transfer processes.

Previous studies have shown that the coupling of electromagnetic phase (OAM) to static matter is rather weak and calls for geometrical fine-tuning of, e.g., an atom into the center of an optical vortex.^25^ For a homogeneous medium, this coupling may even vanish as shown for static chiral polymers.^26,27^ This challenge has already been formulated by Allen et al. in the 90th, and later by Tang and Rosales-Guzmán who discussed communication aspects of OAM transfer.^4,28,29^ On the other hand, the coupling can be enhanced by higher-order coupling terms due to dominating fluctuations, resonances, or longitudinal electromagnetic field components, leading to an avenue for strong light-matter coupling.^22,23,29^ Therefore, we focus on systems with fluctuations tuned by temperature and composition through a chiral/nonchiral phase transition as well as a length scale that can be adapted to the wavelength of the incident structured light. Exactly, chiral phases of liquid crystals (LCs) appear as a model system here.

The chiral phase of nematic LCs shows a repetitive, spiral arrangement of molecules with a pitch length, *p*(*T*, *x*), of the order of the wavelength of visible light, see Figure 1a. *p*(*T*, *x*) depends on temperature and composition, exists only in the chiral phase, and is easily probed by a maximum in the reflectivity. Accordingly, a chiral sample shows a marked difference of color appearance in transmission and reflection of chiral, white light; see Figure 1b. Differential scanning calorimetry (DSC) of LC mixtures is used to evaluate the transitions from the high temperature isotropic to the chiral nematic phase; see Figure 1c,d, cooling and heating.

**Figure 1:**
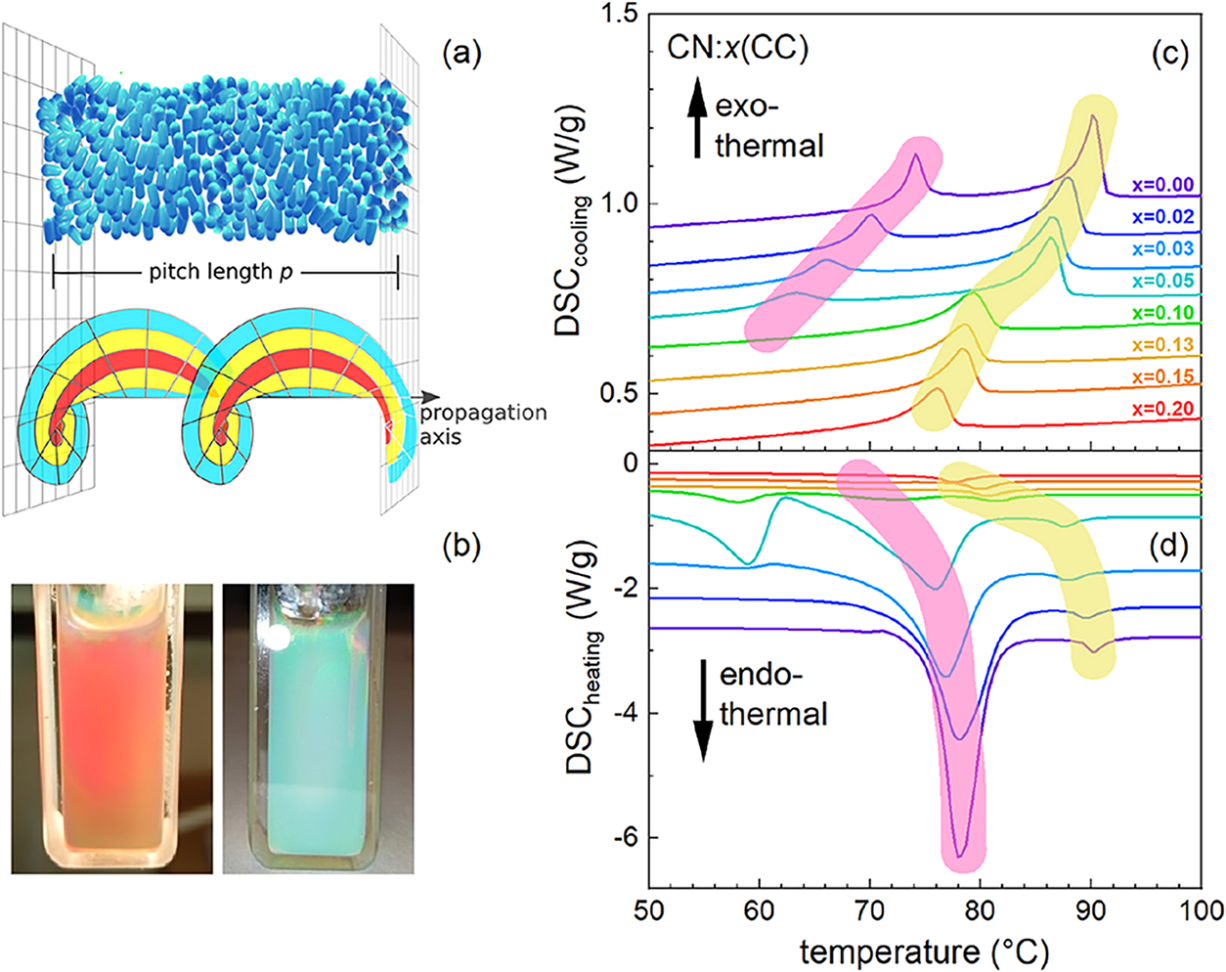
(a) Sketch of molecular orientation in the chiral phase of a LC with the pitch length *p* together with the electrical field evolution of vortex light with the topological charge l = 2; (b) photos of a chiral LC (*x* = 0.2) in a cuvette illuminated by white light from the back and front, respectively. The color difference corresponds to selective reflection of light with λ_exc._ matching LC’s *p*. (c) and (d) DSC of CN:*x*CC during cooling and heating, respectively. Transparent lines connect the respective anomalies, as a guide to the eye.

The effect of temperature and composition on *p*(*T*, *x*) of the chiral phase is due to an interplay of thermal fluctuations versus long-range dipole–dipole interactions,^30^ as well as screening of dipole–dipole versus short-range steric interactions. Increasing the temperature leads to a decrease in the *p*(*T*, *x*). Adding a chiral LC dopant by altering the composition *x* expands *p*(*T*, *x*). Furthermore, *p*(*T*, *x*) can be altered by geometric or optical confinement, i.e., geometric boundary conditions due to anchoring at surfaces, local fields, and strong emitters, e.g., plasmonic effects due to metallic nanoparticles.^9,10^

LCs have large polarizability, pronounced optical nonlinearity^31,32^ and feature birefrigence.^33^ Even light-induced transition phenomena exist.^34^ Therefore, they are perfect candidates to probe the coupling of the OAM and SAM of incident light to chiral fluctuations. Here, the key optical process is iridescence, resembling the behavior of a photonic crystal.^35,36^ Light that resonates in wavelength λ_exc_ with *p*(*T*, *x*) and is in phase with the geometric arrangement of molecules will be localized and selectively reflected, see the fitting phase evolution of light and matter sketched in Figure 1a.

## Experimental Details and Methods

### Chiral Liquid Crystals and Nanoparticles

Mixtures of cholesteryl nonanoate (CN) doped with cholesteryl chloride (CC) denoted as CN:*x*CC with the concentration *x* of CC given by the weight ratio are prepared from powders (Sigma-Aldrich) by melting. For the phase diagram, see Figure S1.

Samples for the measurements are again homogenized by melting the LC mixtures in a cuvette to its isotropic phase and thereafter cooling to the desired temperature in the chiral phase. Mixtures with plasmonic Au nanoparticles of diameter *d* = 20, 50, 100, and 150 nm (Nanochemazone) have been prepared in a similar way. See SI for further information. Such particles are known to enhance Raman scattering.^37^ The rational of our selected particle sizes follows earlier studies^38,39^ that showed peaks in the absorbance at 521 and 533 nm for 20 and 50 nm, respectively. These peaks match with the Laser excitation at 532 nm. Larger NP’s lead to a sudden broadening of the absorbance peak from 80 to 150 nm.

### Generation of Structured Light and Laguerre-Gaussian Laser Beams

Circular polarized light (CPL) OAM beams described by Laguerre-Gaussian (LG) polynomials^4,20^ are generated using *q*-plates^40^ (Zero-Order Vortex Half-Wave Retarders, Thorlabs) adapted to the excitation wavelengths λ_exc_ = 532 nm and λ_exc_ = 633 nm. These plates use an optical spin-flip from CPL to generate OAM with a so-called topological charge l = −4, −3, ..., 4 within the azimuthal phase *e* ^*ilφ*^. They have a very high efficiency of >97% ^40,41^ and a high quality of the phase front, see below. The resulting LG beams are focused to 100 μm on the sample, following a sampling optics with a comparably small opening angle (aperture), see SI for details. In this way, SAM and OAM are not transferred to each other and the averaging of scattering momenta following the scattering process is limited.^41^

In Figure 2a,b, we show the resulting intensity profiles of LG beams with l=4 for λ_exc_ = 532 nm and for λ_exc_ = 633 nm. The intensity is highly structured and exhibits the expected donut shape with a pronounced minimum in the center due to destructive interference and a larger number of rings in the outer region.

**Figure 2:**
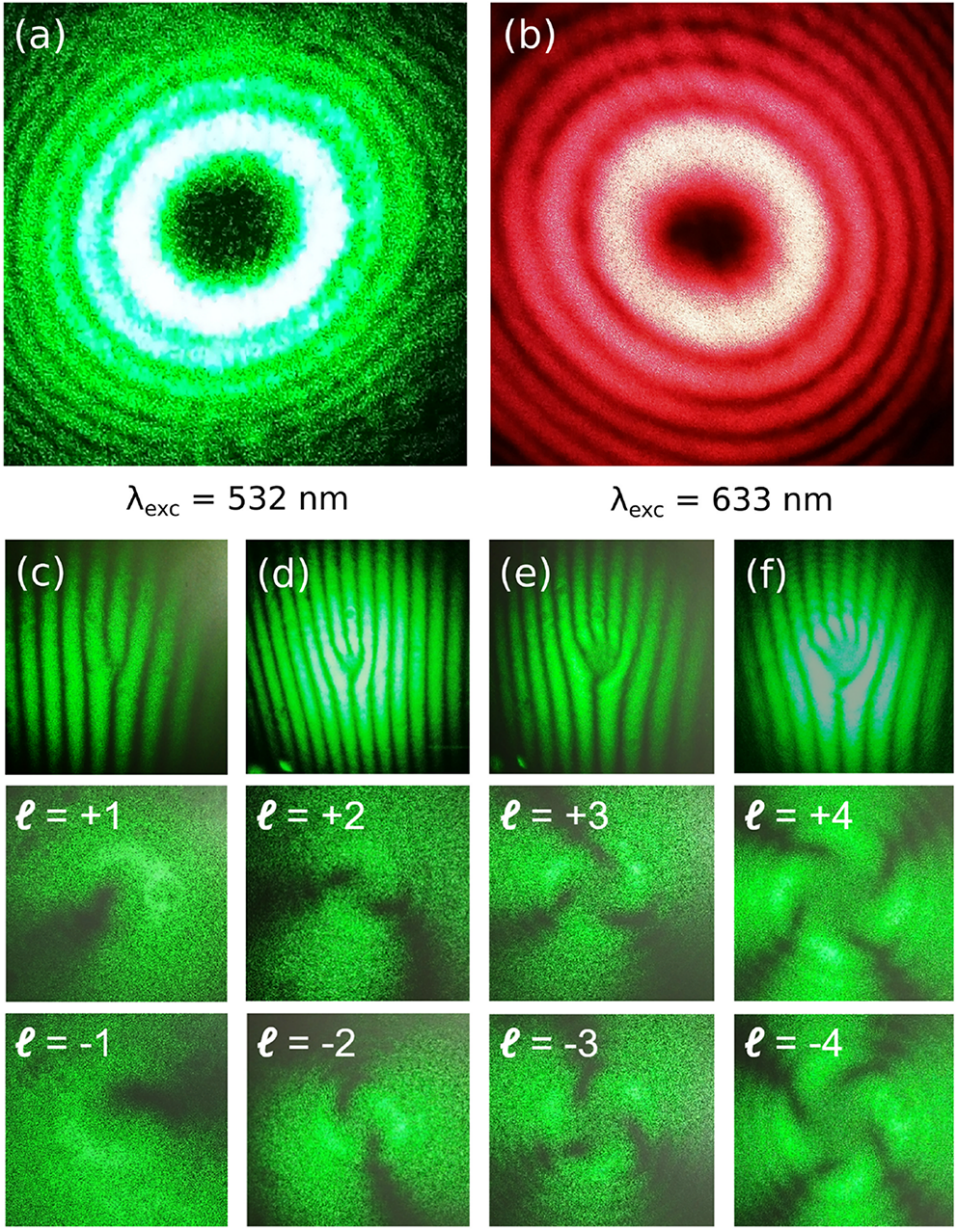
Intensity profile of a light beam carrying an OAM of l=4 and an excitation wavelength of (a) λ_exc_ = 532 nm and (b) λ_exc_ = 633 nm. (c–f) Interference pattern between linearly polarized light and light with an OAM from a Mach–Zehnder interferometer. (top row) Fork-shaped interference pattern with additional emerging streaks according to l=1,2,3,4 ; (middle and bottom row). Interference pattern of OAM light according to its magnitude, l . All of the pictures are photos directly recorded from an observation screen.

Using a Mach–Zehnder interferometer, we characterize the resulting phase front of all optical elements in the beam path. In Figure 2c–f, we show interference patterns between linearly polarized light and LG beams. The topological number, l can be verified according to the streaks emerging from the fork-shaped patterns. The middle and bottom row pictures give respective holograms with the OAM magnitudes as indicated. All pictures show the expected vortex-like features with large contrast, independent of the used OAM. Similar pictures and methodologies for other wavelengths may be taken from the literature.^42,43^

### Experimental Temperature Stages

A Peltier cooler/heater (Belektronig BTC-LAB-A2000) with a homemade setup is used for spectroscopically resolved, selective reflectivity data. Such an experimental setup enables us to determine the temperature regime of iridescence within the chiral nematic phase. The sampling optics leads to an averaging of these dependencies via their opening angle (aperture). All experimental data of reflectivity and Raman scattering are obtained by cooling the samples from the isotropic liquid into the chiral phase.

### Selective Reflectivity

Spectroscopically resolved selective reflectivity is obtained by the difference of two measurements subtracting the intensity of right-handed (RH) CPL from left-handed (LH) CPL, i.e., *I*_LH_–*I*_RH_. The latter setup uses a white light source (Scott KL 1500, incidence angle ≈ 30°) though a polarization filter and λ/4-plate to generate circularly polarized light. Reflected light is collected using a lens with focal lengths of *f* = 50 mm and fiber optics into a mini spectrometer (Hamamatsu, TG series C9405CC). The samples show various iridescent colors as a function of temperature that identify the phase and the pitch length, *p*(*T*). All measurements are performed by initially heating the sample into the isotropic phase and a following cooling process to the desired temperature.

### Characterization by Differential Scanning Calorimetry

Differential scanning calorimetry (DSC) is used to characterize the phase transition of LCs with tailored *p*(*T*) in a thermodynamic way (DSC-3, Mettler-Toledo). The cooling rate is 20 K/min in a dried nitrogen flow of 20 mL/min. For each sample, five heating/cooling cycles were measured, confirming reproducible results. See SI for the *T*, *x* phase diagram.

### Inelastic Light Scattering

Excitation is performed by single frequency lasers with wavelengths of λ_exc_ = 532 nm and λ_exc_ = 633 nm, respectively. The light is guided by various mirrors and passes through various optics and filters. See Figure S2 for a sketch of the setup. A nearly closed aperture limits the laser beam to its paraxial regime, necessary for our experiments with light carrying an OAM.^44^ Depending on the orientation of the λ/4-plate relative to the incident electromagnetic vector field, either the LH CPL or RH CPL is generated. Thereafter, a *q*-plate generates light with an OAM. The laser beam is then focused into the LC sample about 500 μm behind the boundary surface of the sample and the cuvette. This focusing is important as it reduces the scattering from the glass liquid interface and optimizes the intensity due to chiral fluctuations in the LC. A collimator lens with a large focal length of *f* = 250 mm ensures an averaging only over a comparably narrow angle range of the *k*-dependent scattering. The setup for RS experiments in transmission geometry is similar to the backscattering setup, with the only difference that laser light is transmitted through the sample. The laser light is focused inside the sample near the boundary to the window adjacent to the collecting lens.

## Results

In the first part of our study, we monitor light-matter interaction using spectroscopically resolved, chiral reflectivity, I­(λ) = *I*_LH_(λ) – *I*_RH_(λ), with LH and RH being left and right-handed circular polarized light (CPL), respectively. I­(λ) shows a pronounced peak at *p*(*T*, *x*) = λ_res_/*n*, with *n* being the LCs diffraction index. Studies of LCs as a function of composition and temperature are further adapted by adding plasmonic Au nanoparticles that serve as internal light emitters and local field enhancement. All respective data are shown in Figure 3 with numerical values given in Tables S1 and S2.

**Figure 3:**
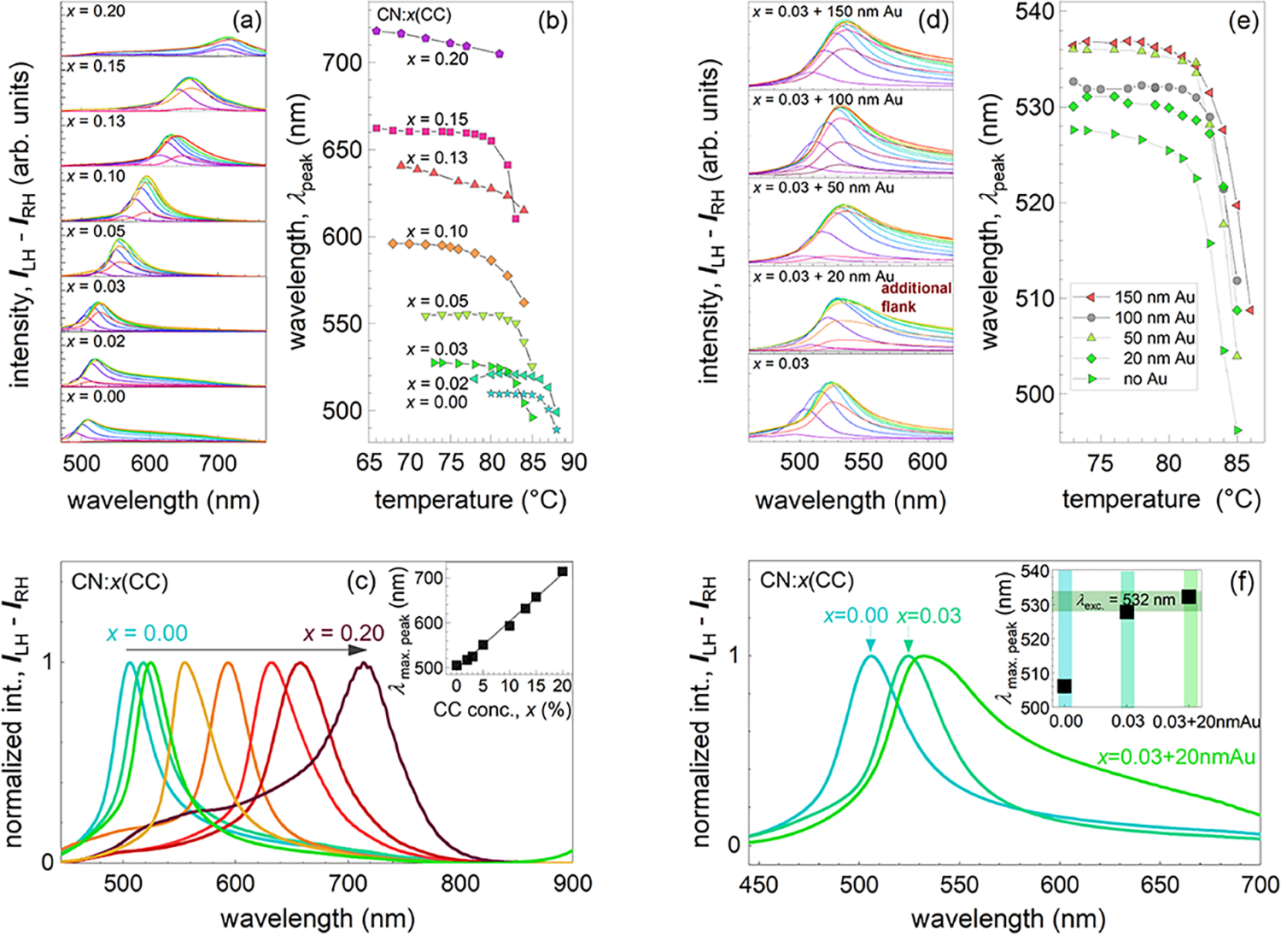
(a, b) Chiral reflectivity, *I*(λ)=*I*_LH_(λ) – *I*_RH_(λ) and peak wavelength of CN:*x*CC as a function of composition and temperature. The temperature sequences of curves in panel (a) correspond to the symbols in panel (b); (c) normalized reflectivity data at the temperature of its maximum height; inset gives the peak wavelength as a function of *x*; (d) chiral reflectivity of *x* = 0.03 with 1% Au nanoparticles of different diameters, from 20 to 150 nm, including a reference sample; (e) corresponding peak wavelength λ_peak_; (f) normalized reflectivity data of the reference sample, CN *x* = 0.00, *x* = 0.03, and *x* = 0.03 with 20 nm Au particles, the inset gives the peak wavelength of these three samples. The bar corresponds to the wavelength of the laser used for the Raman scattering experiment.

In the second part, we present inelastic light scattering that uses the localization of vortex light to amplify an inelastic interaction process, as shown in Figure 4 and in Table S3. As a major outcome, we understand the transfer and control of orbital and spin angular momentum (OAM and SAM) from light to matter due to resonant processes.

**Figure 4:**
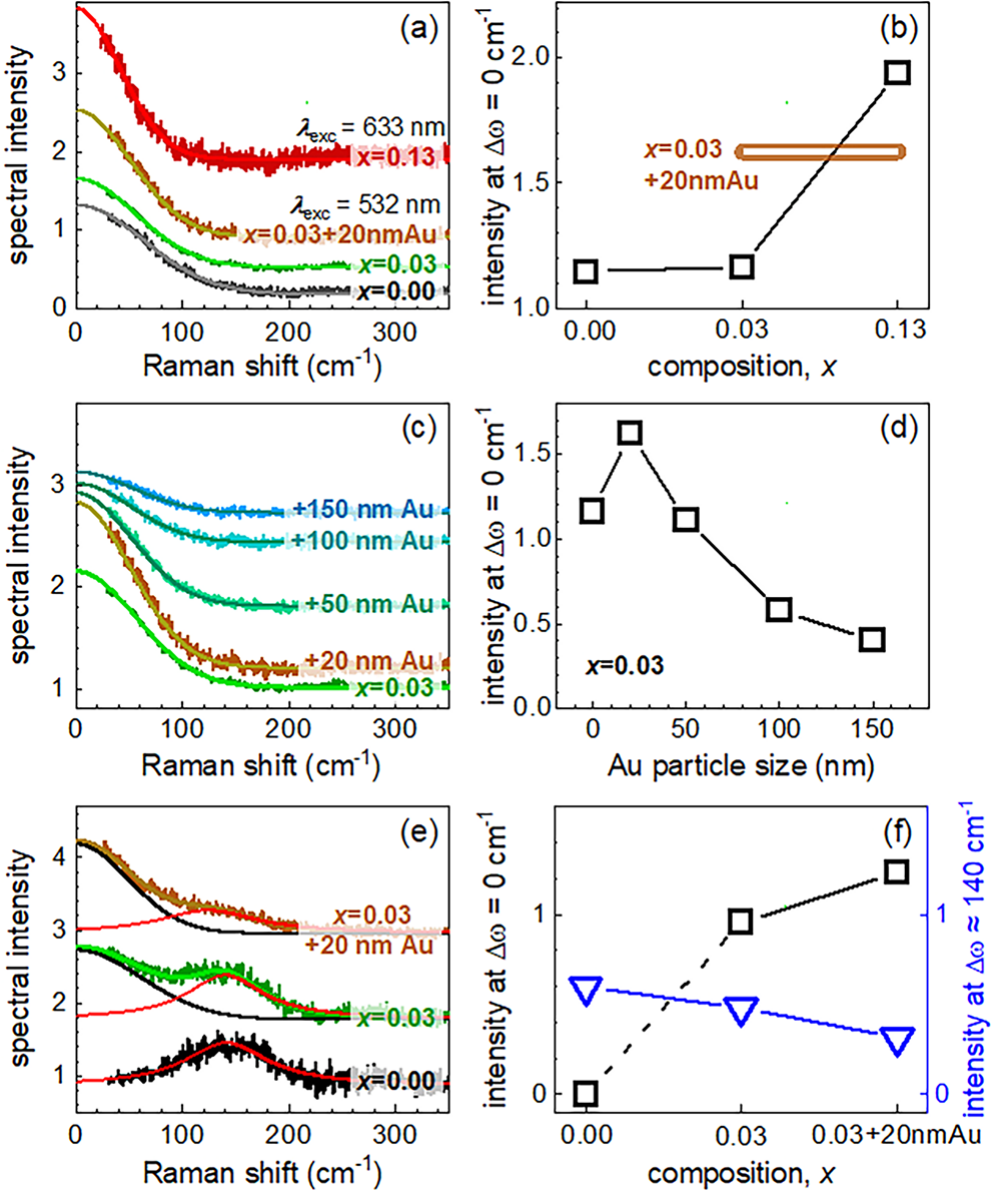
(a) Backscattering RS data for CN:*x*CC with l = 2 divided by data with l = −2 as a function of temperature and *x* = 0.00, 0.03, 0.03 with 20 nm Au and 0.13; (b) respective intensities and an open bar for the Au doped sample; (c) backscattering RS data for *x* = 0.03 with added plasmonic nanoparticles as a function of particles size; (d) respective intensities; (e) transmission RS data for *x* = 0; 0.03, and 0.03 with 20 nm nanoparticles; (f) respective transmission RS intensities. Experimental errors correspond to the symbol size.

### Resonant Chiral Reflectivity and the Phase Diagram of Adapted LC Mixtures

Mixtures of cholesteryl nonanoate (CN) doped with cholesteryl chloride (CC) are well-known for their superior nonlinear optical properties and systematic behavior.^45,46^ Here, we derive the *T*, *x* phase diagram from DSC measurements, see Figure 1c,d and SI. The compositions CN:*x*CC are given by the weight-% of CC, being in the range of 0.0 ≤ *x* ≤ 0.20. The exothermal effect (Figure 1c, marked yellow) shows up upon cooling as latent heat, pointing to a first-order type of phase transition. Consequently, a clear hysteresis of the transition temperature emerges at the homogeneous liquid to LC phase boundary.

The temperature window of the chiral phase shifts and expands to lower temperatures with increasing *x* upon cooling (Figure 1c, marked purple). The latent heat of the lower temperature transitions decreases pointing to a crossover from first order toward a continuous, second order phase transition. For concentrations greater than *x* > 0.1, the low temperature peak vanishes in a continuum. For these mixtures at even lower temperatures, a glass-like transition is observed as a broadened change of slope. Also, the reflectivity data is broadened as shown below. These phenomena are rather complex^47^ and might diffuse our view on the effect of structured light on a highly ordered chiral phase. For a summary of the DSC data with cooling, we refer to Figure S1.

Figure 3a presents detailed reflectivity data of different CN:*x*CC as a function of the detection wavelength and a sequence of temperatures. This forms an envelope of reflectivity functions, in excellent agreement with the DSC data in Figure 1c. In the chiral phase of these LC’s there exist for each composition *x* one temperature with the largest intensity reflectivity peak at a certain wavelength. See Figure S1, showing this temperature within the phase diagram. The data normalized to each maximum in Figure 3c and its peak wavelength, λ_peak_ (see inset) allow to evaluate its dependence on *x*. The peak shows a systematic and linear red shift with increasing CC concentration. The line width of the peak is constant for *x* ≤ 14% and increase for higher *x*. In this concentration range, the peak closes in to the phase boundary; see Figure S1. The observation of a single maximum in chiral reflectivity supports a molecular order of high coherence. The broadening is evidence for a distribution of pitch length for the highest *x*. In the investigated temperature regime, there is no evidence for any superstructure or modulation of chiral order. This is attributed to the temperature history as we cool the sample from the isotropic liquid into the chiral phase.

### Plasmonic Effects on Selective Reflectivity

Adding plasmonic Au nanoparticles to LC’s with *x* = 0.03 (Figure 3d) enhances and broadens the reflectivity maximum in the larger wavelength regime. This is most evident for the smallest-diameter particles (*d* = 20 nm). Both effects add up to a continuous and temperature independent increase with particle diameter; see the plateaus in Figure 3e. In this evolution, the line width of the main maximum does not change that much. A summary and comparison of normalized reflectivity data are given in Figure 3f and its inset. Numerical data are given in Table S2. We notice that doping CN by CC and Au nanoparticles enhances the reflectivity and shifts its maximum exactly to the wavelength of the laser used for RS experiments.

These findings are surprising. First, despite adding nonchiral Au particles, the observed reflectivity conserves the chiral selectivity of the LC, related to *I*(λ) = *I*_LH_(λ) – *I*_RH_(λ). Second, the reflectivity does not show spectral features directly attributed to plasmonic states.^38,48^ We rationalize these observations as an energy transfer from the plasmon state to the LC’s due to their close proximity. A strong light-matter coupling has recently been observed in systems with chiral plasmonic particles.^10^ We highlight that the concentration of the plasmonic nanoparticles is only 1% (weight percentage), as we intend to induce local effects. Accordingly, the evolution of λ_peak_ with *x* is gradual and there is no alteration of the chiral-isotropic phase boundary that shows up as a suppression of *p*(*T*, *x*) and a high temperature cutoff in Figure 3e. We conclude that added Au nanoparticles do not influence the regime of iridescence (73–85 °C) and we derive *T*_max peak_ = (81 ± 1) °C as a set temperature for the RS experiments to be presented further below.

### Inelastic Processes of Angular Momentum with Chiral Matter

Our main research motivation is to observe and enhance the exchange of angular momentum, i.e., information, with matter via resonant, chiral processes. In previous experiments low energy maxima in inelastic RS intensity have been observed using CPL and structured light.^49^ The largest coupling and intensity have been found comparing data with OAM of l = ±2. This OAM corresponds to an evolution of the electromagnetic phase comparable to the orientation of molecules in LC’s, see Figure 1. In the present experiments, we use the same OAM transfer and instead manipulate the resonance conditions via altering *p*(*T*, *x*) and adding plasmonics to the LC. For details of the generation of the OAM, see *Experimental Details and Methods*. The RS setup and respective methodology are found in Figure S2.

Low energy scattering data are presented in Figure 4a,c,e by dividing scattering data with different handedness and OAM states. In this way, SAM/OAM transfer processes are highlighted and OAM independent signals omitted, e.g., fluorescence backgrounds. This approach is analogous to chiral reflectivity *I* = *I*_LH_ – *I*_RH_ and Raman optical activity using CLP.^50^ A division of data has the advantage of avoiding the additional normalization of the data.

Figure 4a shows the dependence of such RS data with CLP and l=± 2 excitation for different samples, with *x* = 0.00, 0.03, and 0.13. The actual temperature of the measurement corresponds to the temperature of the respective maximum in the reflectivity (Figure 3). RS shows a low energy scattering surplus with a Gaussian line shape centered at Δω = 0 cm^–1^. This line shape points to a diffusion-dominated process (Langevin equation) in contrast to the usual Lorentzian line shape related to a finite oscillator lifetime.^51^

RS intensity is related to chiral fluctuations of the LC as it is based on the fluctuation-dissipation theorem.^52,53^ On the other hand, fluctuating energy distributions should be limited to the temperature scale of the sample. Therefore, this novel contribution should have a high energy cutoff with a flat and unity value in the high energy limit, as indeed observed. The drop in intensity for Raman shifts smaller than Δω ≈ 250 cm^–1^ corresponds to 87 °C (*T* = 360 K, 31 meV). The experimental temperatures of 84, 81, and 75 °C, with increasing *x*, are in very reasonable agreement.

Comparing different samples, we notice that the respective intensity depends on the composition of the LC’s. More precisely, this corresponds to a fine-tuning of the maximum in chiral reflectivity to the wavelength of the incident Laser radiation, λ_inc_. With increasing *x*, this can be rationalized by an improved superposition of *p*(*T*, *x*) with λ_inc_ = 532 nm. For *x* = 0.13 the shift of reflectivity to larger wavelengths fits even better to a different laser line, λ_exc_ = 633 nm. The corresponding reflectivity peaks are indeed λ_peak_ = 506 nm for CN, λ_peak_ = 526 nm for *x* = 0.03, and λ_peak_ = 632 nm for *x* = 0.13, respectively. In addition, the line width of the RS maximum shows a systematic narrowing with composition. Increasing *x* leads to fwhm values of 150.8, 132.6, and 98.7 cm^–1^, supporting the improved superposition.

Figure 4c shows the effect of adding Au nanoparticles to *x* = 0.03 in backscattering. This data are also given in Table S3. The sample with 20 nm diameter particles and λ_inc_ = 532 nm shows an enhancement of 42% due to a plasmonic energy transfer to the LC. Larger particles lead to a decrease of RS intensity, see Figure 4d. We attribute this decrease to a reduced efficiency of the resonance due to the larger wavelength shoulder in reflectivity. In Figure 4b a bar marks the intensity of x = 0.03 with 20 nm particles for reference.

The presented RS data in Figure 4a–d are obtained in backscattering geometry. This is not self-evident, as more information can be gained comparing different scattering geometries, in particular transmission with backscattering data. As our experiments are distinguished by a resonance of λ_inc_ with *p*(*T*, *x*), the periodicity of the LC, backscattering, corresponds to a Bragg reflection with a maximum momentum transfer. Thereby, LC’s properties are probed at the boundary of the chiral Brillouin zone. In this context, iridescence has been discussed as localization of light in photonic crystal-like systems.^36,54^ A completely different case is established with laser excitations parallel to the scattered photons given by the transmission geometry. The induced cone of scattered photons selected by the opening angle of the sampling optics experiences only a small momentum transfer. Such scattering events might be dominated by other processes.

Figure 4e shows such transmission geometry experiments on different LC’s. Interestingly, we observed markedly different results. For *x* = 0.0 (CN only), we observe a finite energy Lorentzian mode centered at Δω ≈ 150 cm^–1^. As mentioned above, such a natural line shape is associated with a lifetime limitation of oscillators or quasi-particles. If the different characteristic energies of the Lorentzian and the Gaussian modes are associated with a single, dispersing excitation, they represent an anomalous, optic mode that strongly softens for large momenta. These momenta relate to the inverse of the pitch length, at the Bragg point of the chiral unit cell of the LC. The softening corresponds to the instability of the LC condensing into a chiral phase.^33^ Such excitations usually exist, e.g., in systems with a competition of short-range repulsive and long-range dipolar interactions,^49^ as in polar molecules,^55^ cold atoms in cavities, dipolar quantum gases, and rotons in suprafluid helium.^56^ These interactions lead to strong nonlocal couplings that seem to entangle easily with OAM, i.e., electromagnetic phase evolution.^1^ Liquid crystals driven by structured light fit this scenario.

With *x* increasing to 0.03 wt % and finally adding plasmonic particles, there is a tuning from the spectral features observed with small momenta to the one with large momenta. The finite frequency peak diminishes, and the Gaussian peak at Δω = 0 cm^–1^ is recovered. Figure 4f shows the corresponding increase of the intensities at Δω = 0 cm^–1^. Using the resonant phenomena induced by a fine-tuning of *p*(*T*, *x*) and plasmonic effects, we are able to modify the interaction effects of structured light with the LC in transmission experiments. It is interesting to note that structured light transmitted and reflected by twisted photonic bilayers leads to a conversion of OAM to SAM modes depending on the optic geometry.^57^ These layers are an analogue to twisted bilayer graphene with Moiré patterns.

## Discussion

We achieved two central results in tailoring the response of chiral LC’s to incident structured light. Using a large set of parameters to tune the pitch length given by the composition, temperature, and plasmonic nanoparticles, we modified iridescence and chiral reflectivity. This leads to a well-defined maximum moving in a large spectral range. Thereby, a 2-fold resonance has been achieved, a geometric resonance of λ_inc_ with *p*(*T*, *x*) together with a phase matching of the helical evolution of light (OAM, l = 2) to the chiral molecular orientation.^58^

Identifying this as being crucial to enhance light-matter coupling,^32,42^ we notice an intensity maximum of inelastic scattering in samples with the reflectivity peak precisely tuned to the incident laser wavelength. The observed spectral feature shows a Gaussian line shape due to diffusive chiral fluctuations of the LC. This process is dominated by large momenta from Bragg reflections of the chiral LC’s unit cell.

Varying the scattering angle, comparing backscattering with transmission, allows us to probe the dependence of momentum conservation on the resonance conditions. In less resonant scattering, with a small difference between the pitch length and λ_inc_, the scattering processes really depend on the transfer of the scattering vector. In contrast, enhancing resonance via *p*(*T*, *x*) ≈ λ_inc_ the role of the scattering vector is diminished, spectral features are no more specific, see the upper two curves in Figure 4e. Adding plasmonic particles with the smallest size and strong dilution completely relaxes this conservation. This is rationalized by their properties as small and diluted emitters that transfer energy from the electromagnetic field to single molecules of the LC.

We identify two contrasting effects as a function of particle size. For small diameters, there is a dominance of plasmonic effects that enhance the scattering intensity. For larger particles, comparable to the pitch length itself, the coherence length of chiral order is reduced, and the reflectivity maximum is strongly broadened with a wing to a larger wavelength. This is supported by the previous observation of a broadened plasmonic adsorbance.^38,39^

In the following, we compare our results with other discoveries that could pave the way for applications of structured light in analytics and communication. Many of these effects rely on a specificity in the detection of OAM states, their spatial resolution, and the multiplexing of information based on OAM variation.^59^ They also demonstrate the advantage that multifunctional aspects of hybrid materials and their interactions processes can have.^60^

In theory, OAM enhanced optical activity and chiroptical processes as circular-vortex differential scattering are mainly relevant for materials with magnetic-dipole and electric-quadrupole transitions.^61^ The predicted enhanced sensitivity on the phase properties of the light beams and scattering-angle dependencies^23,61^ are in close resemblance to our observations. Recently, it has been shown that such higher-order transitions with OAM are expected for plasmonic nanoparticles, especially if they are chiral themselves.^10^ However, also longitudinal field components^18,29^ induced by strong focusing of the incident beam or other OAM to SAM conversion processes may be used.^42,57^

Such longitudinal fields and conversion processes are indeed enhanced in systems with strong long-range dipolar interactions,^56^ e.g., for polar molecules^55^ and dedicated metamaterials.^62^ In LC’s, exactly these forces competing with steric forces are essential for the chiral phase formation.^33^ Therefore, the observed strong coupling effects in our tailored CN:*x*CC series are probably also due to the enhanced dipolar interactions,^31^ in addition to the above-discussed geometric and phase resonances of the vortex excitation. Summarizing, the presented adaption of geometries and momentum relaxation in our LC’s leads to a large variability of these liquids that might propose them as a promising component of hybrid materials and being useful for applications of the OAM in momentum and information transfer.

## Summary

Tailoring a liquid crystal series with respect to its optical and light-matter interaction characteristics allowed us, based on a detailed study of the phase diagram using DSC and chiral reflectivity, to enhance the response with respect to structured light. In line with the identification of iridescence and chiral reflectivity being important for the transfer of angular momentum, we optimized a scattering process related to a 2-fold resonance, a geometric resonance of λ_inc_ with *p*(*T*, *x*) leading to iridescence and a matching of the corresponding OAM phase with the LC orientation. Further contributions might come from long-range dipolar interactions in the system that enhance higher-order optical transitions and energy transfer. The optimized light-matter coupling also allowed us to study the scattering momentum dependence as a function of this resonance. Plasmonic nanoparticles further enhance these processes up to 71% in intensity. However, they release momentum conservation. Our study opens a route to use LC in meta- or hybrid materials as a component with high sensitivity for structured light.

## Supplementary Material



## Notes

Experimental setup, procedures, and characterization data for all compounds (PDF)
