# Supplementary material for: Tailoring Momentum and Information Transfer of Structured Light to Adapted Liquid Crystals
Source: ACS Omega. 2025 Dec 1;10(49):60773–80. doi: 10.1021/acsomega.5c09134 (PMC12713437; doi:10.1021/acsomega.5c09134)
Supplement: Supplementary file 1 [file ao5c09134_si_001.pdf]

# Supporting Information

# on

# Tailoring Momentum and Information Transfer of Structured Light to Adapted Liquid Crystals

Silvia Hofmann,† Peter Lemmens,∗,† and Angela Möller‡

†Institute for Condensed Matter Physics, University of Technology Braunschweig, D-38106 Braunschweig, Germany

‡Department of Chemistry, JGU Mainz, D-55128 Mainz, Germany

E-mail: p.lemmens@tu-bs.de

**Outline:**

- **Sample Preparation and Characterization**
- **DSC and the Phase Diagram of CN:xCC**
- **Effect of composition x and additional Au NP's on Reflectivity and Light Scattering Intensity**
- **Low Energy Light Scattering Setup**
- **Variation of Transmitted Laser Power**
- **References**

**Sample Preparation and Characterization**

Mixtures of liquid crystals have been prepared to tailor their pitch length, $p(T,x)$, and optical properties. Cholesteryl nonanoate (CN) and cholesteryl chloride (CC) have been mixed according to the concentration *x* of CC given by the weight ratio. CN is often named as Cholesterylpelargonat [Sigmaaldrich]. It has the empiric composition $C_{36}H_{62}O_2$ and the phase transition from the cholesteric phase to the isotropic liquid phase appears at 90 °C. Powders have been characterized by X-ray scattering [Muellner2022] and dynamic scanning calorimetry (DSC).

Powders were mixed, filled in cuvettes and heated to the liquid phases (100 °C) while stirring. Subsequent cooling and heating steps have been performed during the DSC experiments without further changes of the heat signals following the initial melting (5 cycles).

Gold nanoparticles (Au NP) with diameters ranging from 20 to 150 nm [Nanochemazone] have been used to prepare plasmonic LC mixtures using CN:xCC, with x=0.03, as described above. The particle diameters have been chosen based on the work by [Link1999] and [Jain2006] to give sufficient overlap with the wavelength of the incident laser (532 nm). For 20 and 50 nm NP's maxima at 521 and 533 nm have been previously observed [Link1999]; while larger NP's lead to a sudden broadening of the absorbance peak from 80 to 150 nm. This agrees well with our observation of a broadened reflectivity data of the doped LC's in Figure 3 (d) and (f). Overall, this follows the general concept of enhancing Raman scattering of molecules due to electronic resonances as observed in surface enhanced Raman scattering [Langer2020].

**DSC and the Phase Diagram of CN:xCC**

We use DSC upon cooling (Fig. 1 c) with the maxima positions to establish a temperature-composition phase diagram, see Figure S1. Within the chiral nematic phase the reflectivity shows maxima due to the resonance of the incident wavelength and the composition and temperature dependent pitch length (Fig. 3 a). From these maxima we choose temperatures with its highest intensity leading to a down folding to a two dimensional phase diagram. In a limited concentration range up to x=0.05, the transition temperatures decrease almost linear. For larger concentrations the first order phase transition temperature remains roughly constant.

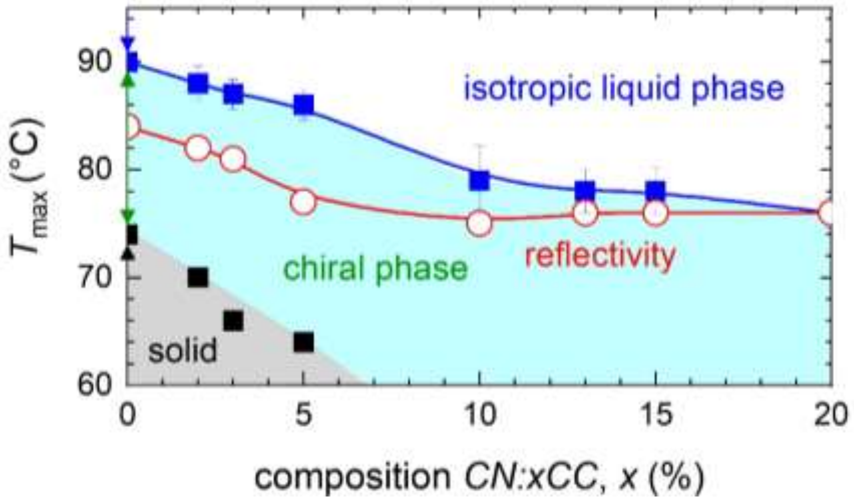


Fig. S1: Temperature maxima of DSC signals as function of composition while cooling the samples. The error bars of the isotropic to chiral phase correspond to the heating-cooling difference. Open symbols denote to the highest intensity maxima in reflectivity. Here experiments are performed in steady state conditions (constant temperatures).

**Effect of Composition x and Additional Au NP's on Reflectivity and Light Scattering Intensity**

In the chiral phase of LC's, we observe resonant and sharp reflection maxima that are due to iridescence of the LC's with a well defined pitch length *p*(T,x). In the next step this length scale is tailored by the composition of the LC's to achieve a maximum overlap with the laser excitation wavelength of inelastic light scattering. In Table S1 detailed data on the reflectivity measurements are given to allow an estimation on the effect of doping.

In the case of x=0.03 and 20-nm NP's the closest proximity to $\lambda_{exc.}$ = 523 nm is gained.

| chiral nem. LC | $T_{irid.}$ | $T_{max.\ peak}$ | $\lambda_{max.\ peak}$ | $FWHM_{max.\ peak}$ |
|---|---|---|---|---|
| CN | (80 – 88) °C | (84 ± 1) °C | (506 ± 3) nm | (31 ± 1) $cm^{-1}$ |
| CN:CC=98:02 | (78 – 88) °C | (82 ± 1) °C | (518 ± 3) nm | (34 ± 1) $cm^{-1}$ |
| CN:CC=97:03 | (73 – 85) °C | (81 ± 1) °C | (526 ± 3) nm | (35 ± 1) $cm^{-1}$ |
| CN:CC=95:05 | (72 – 85) °C | (77 ± 1) °C | (551 ± 3) nm | (35 ± 2) $cm^{-1}$ |
| CN:CC=90:10 | (68 – 84) °C | (75 ± 1) °C | (593 ± 3) nm | (42 ± 2) $cm^{-1}$ |
| CN:CC=87:13 | (69 – 84) °C | (76 ± 1) °C | (632 ± 3) nm | (44 ± 3) $cm^{-1}$ |
| CN:CC=85:15 | (66 – 83) °C | (76 ± 1) °C | (658 ± 4) nm | (55 ± 3) $cm^{-1}$ |
| CN:CC=80:20 | (66 – 81) °C | (76 ± 1) °C | (714 ± 4) nm | (66 ± 4) $cm^{-1}$ |
| CN:CC=97:03+ 1% Au | | | | |
| 20 nm Au | (73 – 85) °C | (81 ± 1) °C | (529 ± 3) nm | (32 ± 1) $cm^{-1}$ |
| 50 nm Au | (73 – 85) °C | (82 ± 1) °C | (535 ± 3) nm | (46 ± 1) $cm^{-1}$ |
| 100 nm Au | (73 – 85) °C | (80 ± 1) °C | (532 ± 3) nm | (38 ± 1) $cm^{-1}$ |
| 150 nm Au | (73 – 86) °C | (81 ± 1) °C | (535 ± 3) nm | (43 ± 1) $cm^{-1}$ |

Table S1: (Reflectivity data) Properties of different chiral nematic CN:xCC compositions and added plasmonic NP obtained from reflectivity measurements. (Second column) T-regime or iridescence with the observation of a maximum in reflectivity; (Third column) $T_{max.\ peak}$ with the largest peak intensity; (fourth row) Wavelength of the maximum peak; (Fifth column) its linewidth, $FWHM_{max.\ peak}$.

| resonant chiral nem. LC | $\lambda_{max.\ peak}$ | $\lambda_{exc.}$ | intensity | linewidth |
|---|---|---|---|---|
| CN:CC=87:13 | 632 nm | 633 nm | 1.93 | 98.7 $cm^{-1}$ |
| CN:CC=97:03 | 526 nm | 532 nm | 1.15 | 132.6 $cm^{-1}$ |
| CN | 506 nm | 532 nm | 1.14 | 150.8 $cm^{-1}$ |

Table S2: (Raman data) Properties of low energy inelastic scattering for different samples and excitation wavelength. The corresponding data from Fig. 4 (a) and (b) is based on a fit to a Gaussian lineshape. Enhanced intensity is correlated with decreasing linewidth of the light scattering maximum.

The intensity and linewidth of the low energy scattering depends on the overlap of the reflectivity maximum with the laser wavelength. The data is analyzed using a Gaussian lineshape with a maximum centered at $\Delta\omega \approx 0\ cm^{-1}$, see Table S2 and S3. A decrease in linewidth is observed together with a maximum in intensity for 20 nm-NP's.

| Au particle size | intensity | linewidth |
|---|---|---|
| 150 nm | 0.40 | 125.6 $cm^{-1}$ |
| 100 nm | 0.58 | 123.1 $cm^{-1}$ |
| 50 nm | 1.11 | 123.3 $cm^{-1}$ |
| 20 nm | 1.63 | 121.1 $cm^{-1}$ |
| 0 nm | 1.15 | 132.6 $cm^{-1}$ |

Table S3: (Raman data) Intensity and linewidth of the low energy inelastic light scattering from CN:CC=97:03+plasmonic Au nanoparticles derived from the data shown in Fig. 4 (d). The sample denoted as "0 nm" corresponds to the reference sample without Au NP's.

**Low Energy Light Scattering Setup**

This setup consists of a triple Raman spectrometer and several optical elements, as shown in Figure S2. The focusing optics of the incident laser and the sampling optics are separated. This allows to reduce elastically scattering light at the glass-liquid interface and sample imperfections. Furthermore, the aperture of the sampling optics, NA=0.08 is rather small due to the large focal length of f= 250 mm. This leads to a smaller integration range of scattering momenta and an optical beampath that can be approximated as paraxial.

The Laser focus of approximately 100 µm diameter has an integrated power of 15 mW (633nm) to 25 mW (532 nm) in backscattering and 26 mW in transmission, respectively. The resulting power density of approximately (3.8 W/mm$^2$) is as factor of 60 smaller than used in other experiments using nonlinear effects [El-Ketara2021, Thodika2025]. In another study a power density threshold of 130 W/mm$^2$ for optically induced transitions has been determined [Olenk1999]. Therefore, we do not consider laser induced effects.

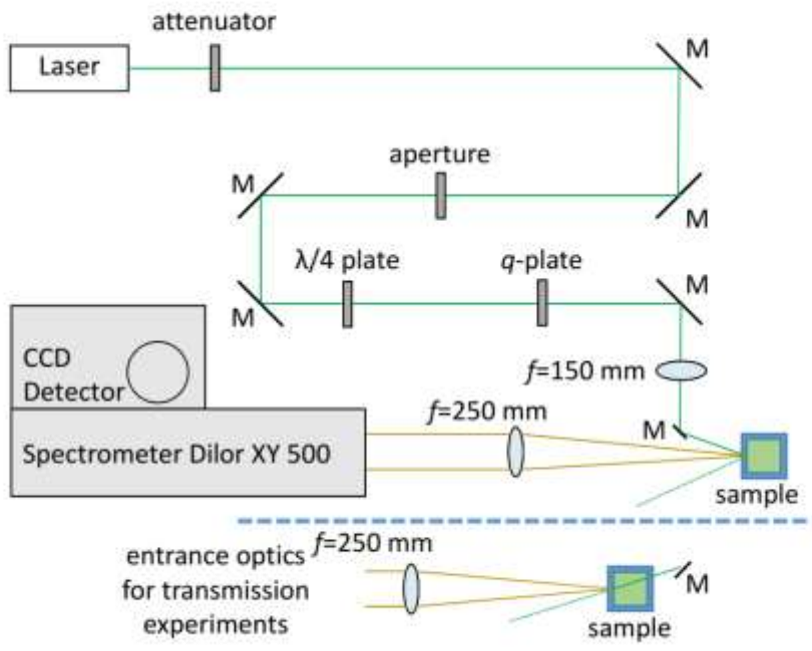


Fig. S2: Sketch of the low energy scattering setup including the sampling optics for backscattering of the sample. Below the dashed line the optics used for transmission experiments is shown. For the latter the mirror (M) for the incident laser is placed behind the sample and the sampling optics is shifted to allow a focusing on the identical point on the front of the sample.

**Variation of Transmitted Laser Power**

As a reference to possible absorption processes, nonlinearities, and losses of Laser power comparing backscattering and transmission experiments, we have performed measurements of the power loss at 532 nm. In Table S4 corresponding data is shown for x=0 and 0.03 as function of ℓ. We noticed a weak variation of the reflected power with ℓ. The transmittance is highest for RH CPL without OAM and lowest for RH CPL with an OAM, ℓ = 2. This supports our observation of an enhanced low energy scattering with finite ℓ. In the resonant case, x=0.03, the differences of power decrease is more pronounced, i.e. from 66% to 52% vs. 52% to 48%, respectively.

| RH CPL+$\ell$ | CN=100 | CN:CC=97:03 | |
|---|---|---|---|
| | $P_{trans.}$ | $P_{trans.}$ | $P_{refl.}$ |
| $\ell = 0$ | $(52.3 \pm 0.8)\%$ | $(65.9 \pm 1.3)\%$ | $(14.1 \pm 0.6)\%$ |
| $\ell = 1$ | $(50.9 \pm 0.8)\%$ | $(62.0 \pm 1.4)\%$ | $(14.2 \pm 0.6)\%$ |
| $\ell = 2$ | $(47.7 \pm 0.9)\%$ | $(52.0 \pm 1.3)\%$ | $(13.1 \pm 0.6)\%$ |
| $\ell = 3$ | $(49.2 \pm 0.9)\%$ | $(55.9 \pm 1.3)\%$ | $(13.2 \pm 0.6)\%$ |
| $\ell = 4$ | $(48.9 \pm 0.8)\%$ | $(54.1 \pm 1.4)\%$ | $(13.4 \pm 0.6)\%$ |

Table S4: Power transmission and reflection of a Laser at 532 nm thought samples with x=0 and 0.03 at T = 84 °C and T = 81 °C, respectively, for different topological index ℓ.

**References:**


[Sigmaaldrich] https://www.sigmaaldrich.com/DE/de/product/aldrich/c78801, molecular weight 526.88, melting point 74-77 °C (lit.), crystalline phase to smectic phase 77.5 °C, smectic phase to cholesteric phase 79 °C, cholesteric phase to isotropic phase 90 °C.

[Muellner2022] S. Müllner, F. Büscher, A. Möller, P. Lemmens, Phys. Rev. Lett. 2022, *129*, 207801. [DOI: 10.1103/PhysRevLett.129.207801]

[Link1999] S. Link and M. A. El-Sayed, J. Phys. Chem 1999, **103**, 4212. [DOI: 10.1021/jp984796o]

[Jain2006] P. K. Jain, K. S. Lee, I. H. El-Sayed, M. A. El-Sayed, J. Phys. Chem. 2006, *110*, 7238. [DOI: 10.1021/jp057170o]

[Langer2020] J. Langer, et al., ACS Nano 2020, *14*, 31. [DOI: 10.1021/acsnano.9b04224]

[Nanochemazone] https://www.nanochemazone.com/product/gold-nanoparticles/, Batch NCZ2901/1223A, 1gr, Purity > 99%, CAS Number: 7440-57-5. The particles have been characterized using dynamic light scattering, UV scattering, and TEM.

[El-Ketara2021] M. El Ketara, H. Kobayashi, and E. Brasselet, Nat. Photonics 2021 *15*, 121. [DOI: 10.1038/s41566-020-00726-2]

[Thodika2025] S. Ch. Thodika and E. Brasselet, Phys. Rev. A 2025, *111*, 023518. [DOI: 10.1103/PhysRevA.111.023518]

[Olenik1999] I. D. Olenik, M. Jazbinšek, and M. Čopič, Phys. Rev. Lett. 1999, *82*, 2103. [DOI: 10.1103/PhysRevLett.82.2103]
